# Taxonomic Reinstatement of the Endemic Chinese Species *Iris thoroldii* (Iridaceae) from *I. potaninii* and Reassessment of *I. zhaoana*

**DOI:** 10.3390/plants12223879

**Published:** 2023-11-16

**Authors:** Eugeny V. Boltenkov

**Affiliations:** Botanical Garden-Institute, Far Eastern Branch, Russian Academy of Sciences, 690024 Vladivostok, Russia; boltenkov@rambler.ru

**Keywords:** China, high-elevation plants, *Iris potaninii* var. *ionantha*, morphology, nomenclature, Qinghai–Tibetan Plateau, taxonomy

## Abstract

*Iris thoroldii* is a perennial herbaceous plant with yellow, blue, or purple flowers. The species is native to the Tibetan Plateau and adjacent areas. In the literature and databases, *I. thoroldii* has long been treated in synonymy with *I. potaninii*. Currently, yellow-flowered plants of *I. thoroldii* are considered *I. potanii*, and blue-flowered plants are considered *I. zhaoana*, a replacement name for *I. potaninii* var. *ionantha*. This study aimed to clarify the taxonomic identity of *I. thoroldii*. A critical examination of original material, herbarium specimens, images of living plants, and the literature has shown *I. thoroldii* to be different from *I. potaninii* in some previously neglected macromorphological traits and to be conspecific with *I. zhaoana*. Thus, *I. thoroldii* is removed here from the synonymy of *I. potaninii* and accepted as a distinct species. This is endemic to China (central Gansu, Qinghai, and northwestern Sichuan provinces, and also Xinjiang Uygur and Tibet autonomous regions) and reaches the highest elevations compared with all other species in the genus *Iris* s.l. A revised taxonomy of *I. thoroldii* is provided, and two color forms, often co-occurring, are accepted: the autonymic yellow-flowered form (including a new synonym *I. tigridia* var. *flavescens* for which a lectotype was designated) and a form with blue or purple colors is proposed here, *I. thoroldii* f. *ionantha*. In addition, images of type specimens and detailed photographs of living plants for easy identification, along with the list of specimens of *I. thoroldii* that were examined, and also, comments on its distribution and habitats are provided.

## 1. Introduction

*Iris* L., when considered in a broad sense, is the largest genus of the family Iridaceae distributed in the north temperate zone [1]. In China, the genus is represented by approximately 60 species [2,3]. In the past five years, each of the in-depth taxonomic studies of *Iris* have led to changes in the scope of delimitation and systematic positions of the Chinese species (e.g., [4,5,6,7,8,9]). In fact, there are still some species of which very little is known, or where their actual identity is poorly understood. For instance, *I. thoroldii* Baker is one such species whose identity has long been misinterpreted or even ignored.

**Figure 1:**
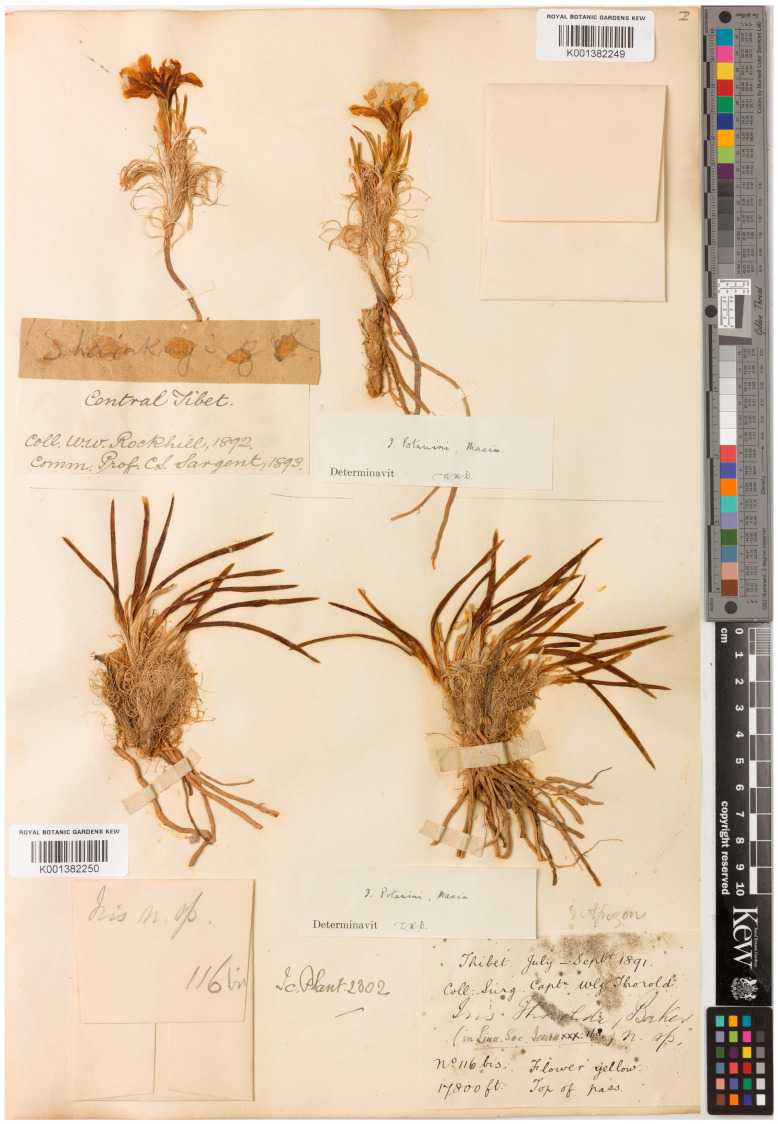
Lectotype of *Iris thoroldii* (K001382250) (reproduced with the consent of the Royal Botanic Gardens, Kew).

When the Kew botanist John Gilbert Baker described *I. thoroldii* from Tibet, China [10], he mentioned that this plant was distinguished by having curled remnants of leaf bases (Figure 1). A plant with this feature is well known from China under a common name, “Curl-sheath iris”, or 卷鞘鸢尾 juan qiao yuanwei in Chinese [2,3,11].

**Figure 2:**
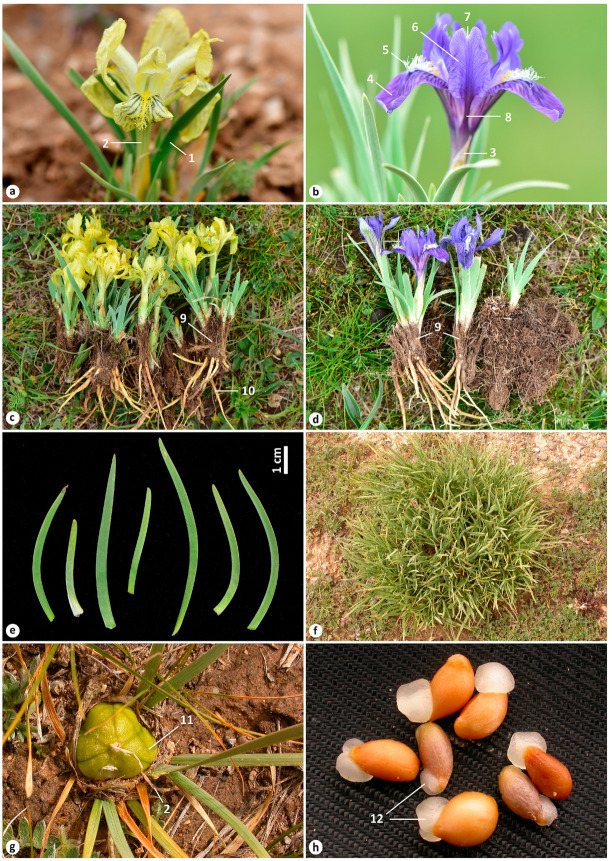
Morphological characters of *Iris thoroldii*: (**a**) yellow-flowered form (Riwoche County, Tibet Autonomous Region, China); (**b**) blue-flowered form; (**c**) habit of yellow-flowered plants; (**d**) habit of blue-flowered plants (Menyuan Hui Autonomous County, Qinghai Province, China); (**e**) leaves (Riwoche County); (**f**) plants in clump; (**g**) in fruiting; (**h**) fresh seeds (Tanggula Mountains, Tibet Autonomous Region, China); (**a–e**) by Xin-Xin Zhu; (**f–h**) by Qinwen Lin. The numbering is as follows: 1, rosette leaf; 2, perianth tube; 3, bract; 4, blade of fall; 5, beard; 6, standard emarginate at the apex; 7, notch; 8, claw; 9, leaf remnants; 10, adventitious root; 11, fruit; 12, aril.

Taxonomically, *I. thoroldii* has been treated in quite different ways. Only few authors have considered *I. thoroldii* an independent species (e.g., [12,13,14,15]). Some important remarks with regard to the taxonomy of *I. thoroldii* were made by Grubov [14]. First, he pointed out that *I. thoroldii* is an endemic species to Chang (or the Tibetan Plateau) and, although closely related to *I. tigridia* Bunge and *I. potaninii* Maxim., is readily distinguished by its obtuse, shortly pointed rosette leaves and by its numerous curled coarse-fibered remnants of leaf bases. Second, *I. thoroldii* is variable in flower color (from pale yellow and yellow, through light and dirty lilac, to purple and dark purple; see Figure 2a–d), and this feature is sometimes observed even within the same clump that consists of plants from different seeds. Grubov concluded that Dykes [16] had erroneously treated *I. thoroldii* as a synonym of *I. potaninii* (see also [17,18,19]) and provided evidence for its recognition as an independent species.

**Figure 3:**
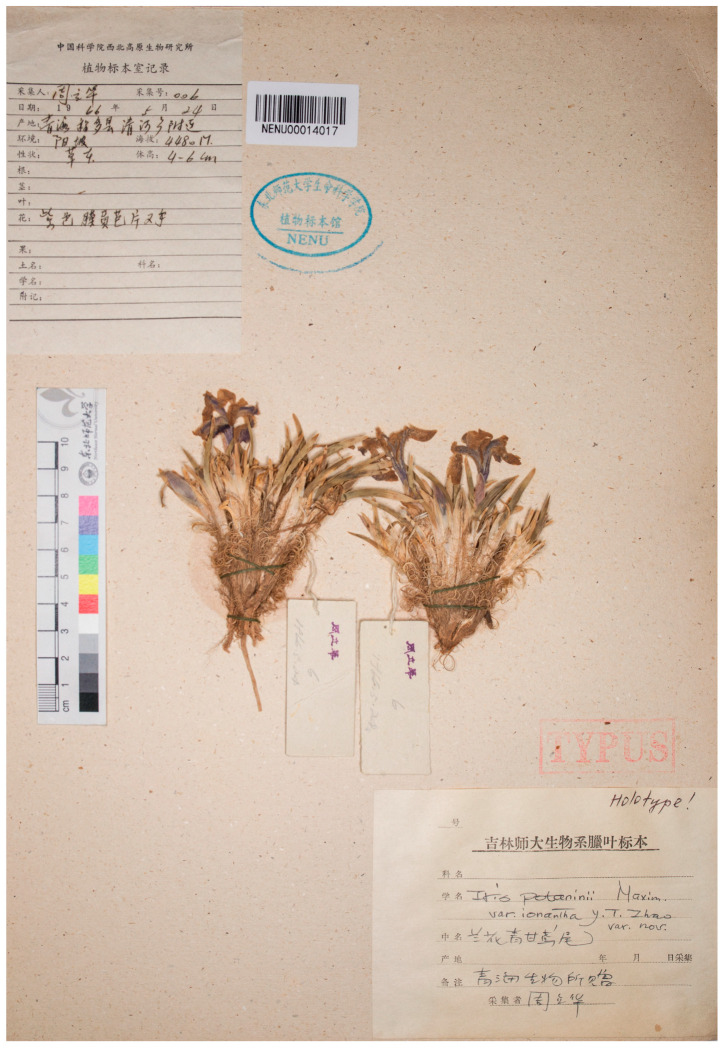
Holotype of *Iris potaninii* var. *ionantha* (NENU00014017) (included with the permission of the curator).

However, Grubov’s opinion was not supported by subsequent authors. Zhao [20] agreed with Dykes [16] and also noted that *I. potaninii* has not only yellow flowers, according to the protologue of the name [21], but also purple flowers, with both color varieties showing the same traits and occurring together in China. Since Dykes [16] combined the yellow- and purple-flowered varieties into a single type, Zhao [20] proposed the name *I. potaninii* var. *ionantha* Y.T.Zhao (Figure 3) for purple-flowered plants. After Zhao [20], *I. thoroldii* has most often been treated as a synonym of *I. potaninii* [2,3,22,23,24,25,26,27,28,29,30,31,32,33,34,35,36,37,38,39] and the Tibetan plants cited under the name *I. potaninii* [32,33,40,41,42,43,44,45,46].

Crespo et al. [25] raised *I. potaninii* var. *ionantha* to the rank of species as *I. zhaoana* M.B.Crespo, Alexeeva & Y.E.Xiao. Following Zhao’s proposal [20], they assumed that only the purple-flowered plants should be included in this new species. However, Crespo et al. [25] did not realize that the purple-flowered species only occurred in western China; they did not see the original material of *I. thoroldii* and provisionally included it in *I. potaninii*, and this issue has not yet been further addressed. As a result, considerable taxonomic confusion remains with regard to the identity of the Tibetan plants to *I. potaninii*. To date, *I. potaninii* and *I. zhaoana* are known to grow together in Tibet as well as in Siberia and Mongolia [26,28,31,34,35,36,39], with the common name of *I. potaninii* being “Curl-sheath iris” [34,37,47].

Recently, it has been suggested, however, that the plants from western China belong to a species that is different from *I. potaninii* (sub *I. potaninii* var. *ionantha*) [9,48]. The true *I. potaninii* has non-curled remnants of leaves, and it is found only in southern Siberia, Mongolia, and northern China [9]. Thus, it has become evident that *I. thoroldii* is erroneously synonymized with *I. potaninii*, as well as the yellow-flowered Tibetan plants are erroneously attributed to *I. potaninii*, while *I. thoroldii* is still ignored, and its color forms have not received proper taxonomic treatment. This study aimed to revise the taxonomy of *I. thoroldii* in order to disentangle the confusion still existing around this name, as described by Baker [10] and determined by its nomenclatural type (Figure 1). The study focuses on the macromorphological characters that are useful for delimiting *I. thoroldii* and *I. potaninii*, while the supporting illustrations may facilitate further identification of these species and contribute to the development of identification keys. A critical examination of relevant academic sources, herbarium specimens, and images of living plants, as well as a complete revision of the *I. thoroldii* nomenclature and analysis of their distribution, have been carried out.

## 2. Materials and Methods

### 2.1. Study Material

The original material of *I. thoroldii* (Figure 1), *I. potaninii* (http://re.herbariumle.ru, accessed on 23 October 2023; see Section 4), and *I. potaninii* var. *ionantha* (Figure 3) was re-investigated personally. During a visit to the English herbaria in March 2014, a search at K was made for the specimens attributed to the name *I. thoroldii*, and the type specimen was found (Figure 1). The type specimen of *I. potaninii* var. *ionantha* was found at NENU (Figure 3) in May 2019. A herbarium-based study of *I. thoroldii* was carried out using 35 specimens (Annex 1), available at LE. These specimens belong to the collections of Nikolai Mikhailovich Przhevalsky from his third (1879–1880) and fourth (1883–1885) journeys to Central Asia, the collection of Vsevolod Ivanovich Roborovsky from the Przhevalsky’s fourth journey to Central Asia, the collection of Grigory Nikolayevich Potanin from his journey to Gansu Province, China (1883–1886), and the collection of Veniamin Fedorovich Ladygin from the Pyotr Kuzmich Kozlov journey to Tibet (1899–1901). All the specimens are accompanied by labels with the printed note “Nota criticae. V. Grubov” and contain Grubov’s handwritten note “*Iris Thoroldii* Baker, 5 March [19]66” (see https://en.herbariumle.ru/, accessed on 23 October 2023). The herbarium codes are according to *Index Herbariorum* [49].

Also, more than 200 specimens of *I. thoroldii* were searched through images available in the databases [50,51], which represent collective data from the Chinese herbaria (BJFC, BNU, HNWP, IBSC, KUN, LZD, NAS, PE, and XJA). The herbarium specimens were identified on the basis of my own experience in dealing with *Iris* species. A complete list of these specimens is provided in Annex 1.

### 2.2. Morphological Data

In order to clarify the differences between *I. thoroldii* and *I. potaninii*, 35 qualitative and quantitative macromorphological characters were selected: (1) rhizome shape, (2) adventitious root shape, (3) rosette leaf shape, (4) rosette leaf apex shape, (5) rosette leaf texture, (6) rosette leaf length (measured from the base to the apex of the longest rosette leaf), (7) rosette leaf width (measured at the broadest part of the broadest rosette leaf), (8) leaf remnants’ shape, (9) leaf remnants’ height, (10) stem height (measured from the base of the flowering stem to the base of the outer bract), (11) number of stem leaves, (12) stem leaf length (measured from the base to the apex of the upper stem leaf), (13) stem leaf shape, (14) number of bracts, (15) bract shape, (16) bract texture, (17) bract length (measured from the base to the apex of the outer bract), (18) pedicel length, (19) perianth tube length (measured from the ovary apex to the base of fall, i.e., outer perianth segment), (20) number of flowers, (21) flower color, (22) flower diameter, (23) blade of fall shape, (24) fall length, (25) fall width, (26) standard (i.e., inner perianth segment) shape, (27) standard length, (28) standard width, (29) fruit shape, (30) fruit length, (31) fruit width, (32) seed shape, (33) seed color, (34) seed length, and (35) seed width. The morphological description of *I. thoroldii* was based on a direct examination of the herbarium specimens from K, LE (Annex 1), and NENU, as well as on the images of well-developed plants in flowering and fruiting stages available in the virtual herbaria [50,51] (Annex 1). I incorporated the morphological data of *I. potaninii* from a recent study [9] and added information on the leaf remnants’ length, number of stem leaves, stem leaf shape, bract shape, flower diameter, and the morphology of fall, standard, and seed obtained as a result of extensive field investigations and thorough examination of specimens from LE and VBGI. The terminology used in the descriptions was based on reference [52]. For the seed morphology, the material from the specimen of *I. potaninii* deposited at LE (Khuvsgul region, Mongolia, 15 km northeast of Urd-daba Pass, 23 July 1972, E. Kukn 248; seeds were measured using an Absolute Digimatic digital caliper, Mitutoyo, Aurora, IL, USA, to an accuracy of 0.1 mm) and an image of *I. thoroldii* (https://www.cvh.ac.cn/spms/detail.php?id=dd49951a, accessed on 23 October 2023; all were measured using AxioVision, version 4.8 (Carl Zeiss, Oberkochen, Germany)) were used.

### 2.3. Taxonomy and Distribution

Here, the conservative taxonomy of *Iris* was used [1,2,3,14,16,23,53]. For the taxonomy, the *Shenzhen Code* (hereafter ICN, [54]) was consulted. The International Plant Names Index (hereafter, IPNI [55]) was also consulted for the nomenclature. In the case of disagreement on the infraspecific rank at which a name should be accepted, I followed Brummitt [56].

In the taxonomic treatment section (see below), I extracted the information on distribution of *I. thoroldii* from the herbarium specimens (see Annex 1) and the databases [28,35,46] where plants of this species are represented, mainly under the names *I. potaninii, I. potaninii* var. *ionantha,* and *I. zhaoana*. I also used the information provided in reference (sub *I. potaninii*) [40] (p. 289), which is recognized here as a reliable source.

## 3. Results

**Figure 4:**
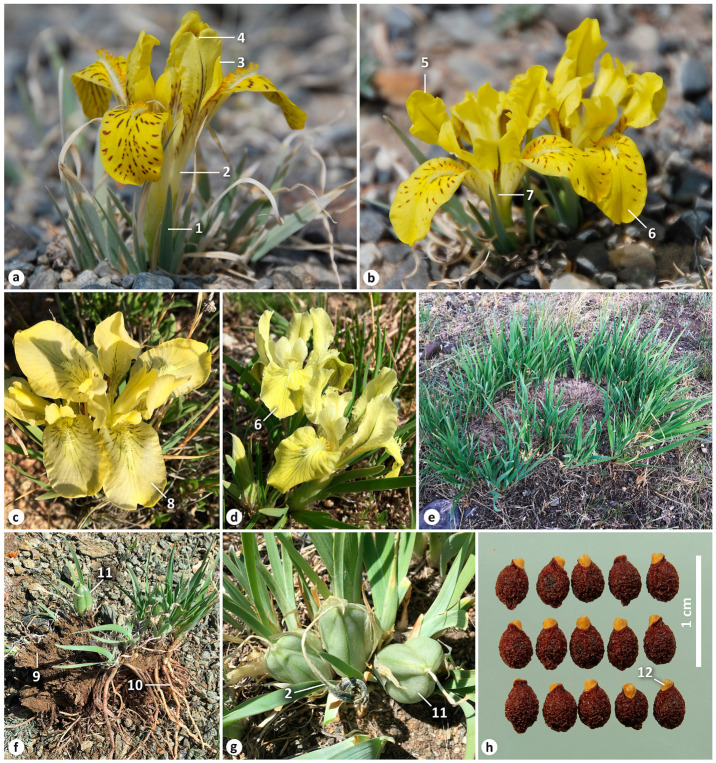
Morphological characteristics of *Iris potaninii*: (**a–d**) flower variability ((**a**,**b**) Tolbo, Bayan-Ölgiy Aimag, Mongolia; (**c**) eastern bank of Lake Gusinoye, Buryatia, Russia; (**d**) Adon-Chelon, Zabaykalsky Krai, Russia); (**e**) plants in clump; (**f**) habit in fruiting ((**e**,**f**) vicinities of Chagan-Uzun, Altai Republic, Russia); (**g**) in fruiting (southern bank of Lake Zun-Torey, Zabaykalsky Krai, Russia); (**h**) seeds; (**a,b**) by Petr Kosachev; (**c–h**) by the author. The numbering is as follows: 1, rosette leaf; 2, perianth tube; 3, standard emarginate at the apex; 4, notch; 5, standard rounded at the apex; 6, blade of fall rounded at the apex; 7, claw; 8, blade of fall emarginate at the apex; 9, leaf remnants; 10, adventitious root; 11, fruit; 12, aril.

A morphological comparison of *I. thoroldii* and *I. potaninii* is presented in Table 1 (also see Table S1 and Figure 2 and Figure 4). Both species share many features, including the following: The rhizome is shortened, weakly branching. Adventitious roots extend from the base of the stem; these are long, with thick upper and thinner lower parts, wrinkled transverse patterns (contractile), few lateral roots, and are yellowish white in color (Figure 2c,d and Figure 4f). Rosette leaves are aggregated in a dense tuft, with the base surrounded by a few papery leaves, narrow-linear, finely ribbed, with waxy coating, and are 0.1–0.5 cm wide. In the wild, the rosette bears straight fibrous remnants of leaf bases, not emerging above ground (Figure 2c,d and Figure 4f). Flowering stems are not emerging above ground; stem leaves are sheath-like and lanceolate, with two bracts, membranous, lanceolate, acute at the apex and without bracteoles. Flowers are solitary, 2–5.5 cm in diameter. Yellow flowers are variable in the color intensity of broken lines of the blade of falls, up to a complete absence of the lines (Figure 2a and Figure 4a–d; also see https://ppbc.iplant.cn/tu/8363242, accessed on 23 October 2023). Flowers are also variable in the shape of falls and standards, having a notch at the apex (emarginate) or being rounded (Figure 2b and Figure 4a–d; also see https://www.inaturalist.org/observations/168065547, accessed on 23 October 2023). The falls have a beard of white, yellow-tipped hairs (Figure 2b and Figure 4a–d); the standards are abruptly narrowed into a narrow claw (Figure 2b and Figure 4b). Fruits are always borne at the soil surface or hidden at the base of rosette leaves and are elliptical, with an obtuse apex and a short beak, which is the proximal part of the dried remnant of the perianth tube (Figure 2g and Figure 4g). Seeds are arillate, brown, glossy, with wrinkled testa (Figure 2h and Figure 4h). Both species often form large colonies or clumps (Figure 2f and Figure 4e).

Morphologically, *I*. *thoroldii* is distinguished from *I*. *potaninii* by its vertical rhizome; by its rosette leaves, which are soft, obtuse, or acute at the apex (Figure 2e) (vs. tough and narrowly acute, Figure 4a); by having longer remnants of leaves, curled after drying (Figure 1 and Figure 3) (vs. always uncurled); by a longer flowering stem, up to 6 cm long (vs. up to 2.5 cm long); by more frequently with 1–2 stem leaves, which are acute at the apex (vs. with three stem leaves that are acuminate); by its bracts being more frequently longer than the perianth tube; by a slightly longer pedicel (vs. extremely short, less than 0.2 cm); by a longer perianth tube; by obovate falls with purple lines (Figure 2a,b) (vs. lingulate falls with brownish lines, Figure 4a–c) and oblanceolate standards (Figure 2b) (vs. obovate, Figure 4c); and by a slightly larger aril (Figure 2h). The variability in the flower color of *I. thoroldii* can be observed within the same locality (see https://ppbc.iplant.cn/tu/9267464, accessed on 23 October 2023) or even clump (see https://ppbc.iplant.cn/tu/9267465, accessed on 23 October 2023). Thus, a yellow and blue-purple color of flowers is a variation observed only in *I. thoroldii*, whereas only a yellow color is characteristic of *I. potaninii*.

## 4. Discussion

### 4.1. Taxonomic Delineation between Iris thoroldii and I. potaninii

Currently, *I. thoroldii* is treated as a synonym of *I. potaninii* [26,27,28,29,30,31,32,33,34,35,36,37,38,39]. However, the differences between them, listed in Table 1, are sufficient to distinguish *I. thoroldii* as a distinct species. In particular, the major difference is the shape of the leaf remnants after drying: they are curled in *I. thoroldii* (Figure 1 and Figure 3) and always straight in *I. potaninii*. The diagnosis can be made even when using specimens without additional organs (e.g., rhizome, rosette leaves, flowering stem, and seed). Plants with curled remnants of leaves are widespread in the Tibetan Plateau and adjacent areas (central Gansu, Qinghai, and northwestern Sichuan provinces, and also Tibet and Xinjiang Uygur autonomous regions, China; see the Taxonomic Treatment section). Thus, the question as to whether the name *I. thoroldii* is a synonym of *I. potaninii* or not has an obvious answer.

Originally, *I. potaninii* was described in 1880 by Carl Johann Maximowicz from Siberia (Russia) on the basis of the specimens collected by Nikolai Turczaninow from Transbaikal (LE01010785, lectotype [9]; LE01010787, LE01010788, and LE01010790), those collected by Alexander von Bunge from the Charysh River (i.e., Altai Republic; LE01010786 and LE01010789), and Potanin’s specimen from Mongolia (LE01011517) [21]. After a comprehensive morphological and genetic characterization, it has been placed in the unispecific *I*. ser. *Potaninia* Doronkin belonging to *I*. sect. *Psammiris* (Spach) J.J.Taylor [9]. It is worth noting here that *I. potaninii* is distributed in steppe patches of the southern Siberian mountain systems in Russia, in northern China (northeastern and western Inner Mongolia, northwestern Heilongjiang Province, and the northern Ningxia Hui Autonomous Region), and in Mongolia at elevations of 550–2800 m [9].

In the journey from Leh (Ladakh Region, India) across Tibet eastward, undertaken by Captain Hamilton Bower in mid-July 1891, William Grant Thorold, Surgeon Captain of the Indian Medical Service, gathered a botanical collection [57]. He collected most of the plants in the Tibetan Plateau (or Chang) west and north of Lhasa and found very few of them northeast of Lhasa [10,58]. The Thorold’s collection was granted to Kew and then treated by William Hemsley [10]. *Iris thoroldii* was described by Baker in Hemsley’s study [10] (p. 105), but, in fact, the taxonomic history of the species had begun six years before.

**Figure 5:**
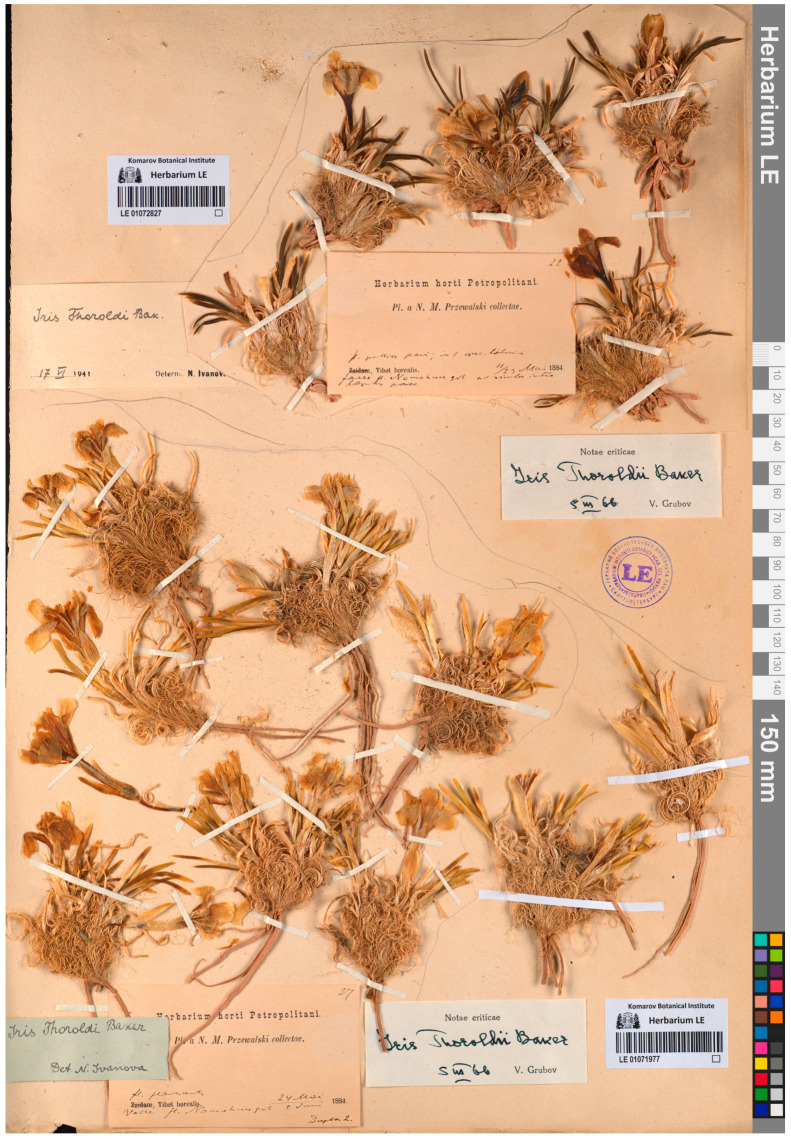
Lectotype of *Iris tigridia* var. *flavescens* (LE01071977) (included with the permission of the curator).

During a critical analysis of the literature, I found a name that is overlooked by botanists, *I. tigridia* var. *flavescens* Maxim. ex Przew. and informed the IPNI about it. This name was described by Przhevalsky on the basis of the specimens from his fourth journey to Central Asia as follows: “...sinii i palevyi kasatiki [“blue and pale-yellow irises”, originally in Russian] (*Iris tigridia*, *I. tigridia* var. *fluvescens*)” [59] (p. 163). This collection, gathered from the tectonic depression Qaidam in the northeastern Tibetan Plateau (Haixi Mongol and Tibetan Autonomous Prefecture, Qinghai Province, China) in early June 1884, was treated by Maximowicz, who identified all the plants [59]. The specimens of *I. tigridia* var. *flavescens* found at LE (LE01071977 and LE01072826; Figure 5) are identical to the original material of *I. thoroldii* (Figure 1), because these plants are yellow-flowered and have curled remnants of leaves.

After being described, the taxonomic identity of *I. thoroldii* was neglected or misunderstood. Most likely Wright, in reference [60] (p. 83) also known as *Index Florae Sinensis*, was the first to have combined Potanin’s gathering from Gansu Province (K000499074, the right-hand plant; see https://powo.science.kew.org/taxon/urn:lsid:ipni.org:names:438985-1, accessed on 23 October 2023) with *I. potaninii* from Dahuria, Altai, and Mongolia. Moreover, Diels [61] (p. 249) treated the collection of Wilhelm Filchner from his journey to Tibet in 1903–1905 and cited the gathering “Tibet: Lab-ts’e, mit Blüten am 7. Juli (Nr. 99)” under the name *I. potaninii*. He noted that *I. thoroldii* was a variety of *I. potaninii* from southern Tibet. Thus, this statement of similarity between *I. thoroldii* and *I. potaninii* by Diels was enough to refuse Baker’s name.

Dykes [16,17], following Diels [61], considered *I. thoroldii* a synonym of *I. potaninii*. Based on Przhevalsky’s collection, he also noted that the purple-flowered form occurred among the yellow-flowered plants of *I. potaninii* from northern Tibet [62,63], which was refuted by Grubov [14], who recognized that the Tibetan plants with yellow and blue-purple flowers belong to *I. thoroldii*.

Despite Grubov’s remarks, Zhao [20] accepted Dykes’ concept of *I. potaninii* and proposed *I. potaninii* var. *ionantha* for the purple-flowered Tibetan plants. Subsequently, botanists also erroneously suggested *I. potaninii* to have not only yellow but also purple flowers; they kept combining *I. thoroldii* and *I. potaninii* and treated the plants from western China as color varieties of *I. potaninii*, i.e., *I. potaninii* var. *potaninii* with yellow flowers and *I. potaninii* var. *ionantha* with blue or purple flowers [3,22,23,24,40,64,65,66,67]. Furthermore, the name *I. zhaoana* was proposed to replace *I. potaninii* var. *ionantha* and was included in *I*. sect. *Pseudoregelia* ser. *Tigridiae* Doronkin [25]. As a consequence, two erroneous views have been stated: (1) *I. potaninii* and *I. zhaoana* grow together on the Tibetan Plateau, and (2) *I. zhaoana* is native to Siberia and Mongolia [26,28,31,34,35,36,39].

However, Thorold’s specimen (Figure 1) indicates something that has been overlooked. It is clear that the original material of *I. thoroldii* represents the same species currently known under the name *I. zhaoana* (Figure 3). Thus, Baker’s name *I. thoroldii* is reinstated here for the yellow-flowered and blue-flowered Tibetan plants with curled remnants of leaves. As confirmed in the present study, based on an analysis of the herbarium specimens (see Annex 1), images of living plants [28,35], and the literature [14,59,65], some populations of *I. thoroldii* in the wild are represented by the yellow-flowered form only and are fairly uniform in their coloration, while others are represented by a mixture of the yellow and blue-purple forms.

Moreover, recent molecular data have strongly supported the assumptions that the plants from western China (sub *I. potaninii* var. *ionantha*) [9,48,53] represent a species that is distinct from *I. potaninii*, that this Chinese species belongs to *I*. sect. *Pseudoregelia* rather than to *I*. sect. *Psammiris* as true *I. potaninii*, and that *I*. ser. *Tigridiae* (with *I. tigridia*) is a unispecific group belonging to *I*. sect. *Psammiris*. A thesis reporting on a genetic study of *I. potaninii* from China [68] deserves a special mention here. An analysis of 128 individuals from 15 populations has shown that the plants from the Hulunbuir Plateau (northeastern Inner Mongolia, China), where true *I. potaninii* occur according to the reference [9], exhibit clear genetic differentiation from the plants collected in the Tibetan Plateau, where *I. thoroldii* is distributed according to Grubov [14] and to the present study. Furthermore, it has provided evidence that the Yushu area is the center of distribution of Tibetan plants’ genetic diversity.

### 4.2. Taxonomic Treatment

The results of this study, based on morphological data, show that the Tibetan plants traditionally identified as *I. potaninii* and currently identified as *I. zhaoana* should be considered a distinct species because of the well-defined morphological traits discriminating them from *I. potaninii*. It is undoubtedly *I. thoroldii* whose original material (Figure 1) is identical to *I. potaninii* var. *ionantha* (≡*I. zhaoana*; Figure 3), except in terms of flower color. Thus, to follow the nomenclatural principles [54], the earliest legitimate name of the taxon, *I. thoroldii*, is restored here (Art. 11.4 of the ICN).

Furthermore, while describing *I. thoroldii*, Baker cited a single gathering collected by Thorold at the “top of the pass, at 17,800 ft.” [10]. Grubov [14] (p. 100) cited the specimen from K, which was collected from Changtang, a high-altitude plateau in western and northern Tibet, including the southeastern regions of Ladakh, as follows: “Top of the pass, at 17,800 ft. [5430 m], 116 bis, 1891–Thorold, typus!”. At K, I found a specimen (K001382250) that belongs to the original material of *I. thoroldii*. It consists of two plants without flowering stems (Figure 1). However, the protologue of *I. thoroldii* includes the description of a flowering stem and a flower. This may suggest that specimen K001382250 is a part of the original material, and Baker used uncited specimens when composing the description of *I. thoroldii*. According to Art. 9.4 of the ICN, original material comprises elements such as specimens and unpublished illustrations that were available to the author prior to, or at the time of, preparation of the description and diagnosis. In the same study on *I. thoroldii*, Hemsley [10] (p. 139) cited the specimen in flowering collected by Woodville Rockhill as follows: “Sharaknyi-gol, hill-slope at 13,800 ft. Lat. N. 35°50′, long. E. 93°27′”. He indicated the unpublished illustrations as follows: “Hook. Ic. Plant. ined.” (i.e., [12]). Also, he noted that *I. thoroldii* had been described from the specimens collected by Thorold, whereas Rockhill’s specimens contain flowers [10]. Thorold’s gathering (K001382250) and Rockhill’s gathering (K001382249) were actually mounted on a single herbarium sheet (Figure 1). Thus, in citing “typus”, Grubov designated the specimen in K (K001382250) as the lectotype of *I. thoroldii*, satisfying the requirements of ICN (see Art. 9.1, Note 1).

The present study has confirmed that two main colors, yellow and blue, are observed in *I. thoroldii*. However, these are not important for the segregation of *I. thoroldii* into subspecies or varieties, as the differences have no relationship with distribution. According to Brummitt [56], a form should be a variant of conspicuous morphological feature occurring together with other variants of this feature in mixed populations. Thus, two forms of *I. thoroldii* are accepted here: the autonymic yellow-flowered form (Figure 2a,c), as indicated in the protologue (“… *floribus minoribus sulphureis* …”) [10], and the form with a blue or purple color (Figure 2b,d), which was described by Zhao as *I. potaninii* var. *ionantha* [20]. A name at a new rank is proposed here for the form of *I. thoroldii* with blue or purple color (see Art. 32A.1 of the ICN).

The information on both forms, with full nomenclature citations and main findings on the distribution and habitat of *I. thoroldii*, is provided below. The accepted names are highlighted in bold.

***Iris thoroldii*** Baker, J. Linn. Soc., Bot. 30: 118, 1894 (“*thoroldi*”).—Protologue citation: “Top of the pass, at 17.800 ft.”.—Lectotype (designated by Grubov [14] (p. 100) as “typus”): [China, Tibet Autonomous Region], [Handwritten by J.G. Baker]: *Iris Thoroldi*, Baker n. sp.; [Handwritten by W.G. Thorold]: Thibet, top of pass, 17,800 ft., flower yellow, July–September 1891, Surg. Capt. *W.G. Thorold 116*, *bis* (K001382250!).—Figure 1.—Further original material: [China, Qinghai Province, Haixi Mongol and Tibetan Autonomous Prefecture] Sharaknyi-gol, [hill-slope at 13,800 ft.], [35°50′ N 93°27′ E], [29 May] 1892, *W.W. Rockhill s.n*. (K001382249!).—Figure 1.—*Iris potaninii* auct. non-Maxim. [2,3,11,16,17,18,19,22,23,24,40,41,42,43,44,45,60,61,62,63,64,65,66,67,68,69,70,71].

***Iris thoroldii*** f. ***thoroldii***

= *Iris tigridia* var. *flavescens* Maxim. ex Przew., Fourth Trip Centr. Asia: 163, 1888 (“*fluvescens*”), ***syn. nov*.**—Protologue citation: “[Plateau of northeastern Tibet, Przhevalsky]”.—Lectotype (designated here): [China, Qinghai Province, Haixi Mongolian and Tibetan Autonomous Prefecture] Valle fl. Nomohungol, fl. flavescentis, 24 May/5 June 1884, *N.M. Przhevalsky s.n*. (LE01071977!).—Figure 5.—Further original material: Fauce fl. Nomohungol, lecta ex terra selata nost frigis–23°, fl. flavescentis, 23 May/4 June 1884, *N.M. Przhevalsky s.n*. (LE01072826!).—http://rr.herbariumle.ru/01072826 (accessed on 23 October 2023).

***Iris thoroldii*** f. ***ionantha*** (Zhao) Bolt., ***comb. et stat. nov***. ≡ *I. potaninii* var. *ionantha* Y.T.Zhao, Acta Phytotax. Sin. 18(1): 59, 1980 (basionym) ≡ *I. zhaoana* M.B.Crespo, Alexeeva & Y.E.Xiao, Phytotaxa 470(4): 284, 2020.—Protologue citation: “Qinghai: Chengdo, 1966.5.24, L.H. Zhou 006”.—Holotype: [China] Qinghai, Chindu, 24 May 1966, *L.H. Zhou 6* (NENU00014017!).—Figure 3.

Illustrations of *I. thoroldii*: [2] (p. 160), [12], [14] (t. 6), [15] (t. 87), [16] (f. 19), [22] (t. 326), [25] (f. 1B), [40] (t. 46, f. 2–3), [41] (p. 536, f. 2–3), [45] (p. 75), [66] (f. 487), [67] (f. 358), [69] (t. 607), [70] (t. 122).

### 4.3. Notes on the Distribution and Habitat

*Iris thoroldii* is endemic to China, distributed in the Tibetan Plateau and adjacent areas, including central Gansu Province (Jinchang and Zhangye prefecture-level cities), Qinghai Province (Golog Tibetan Autonomous Prefecture: Banma, Darlag, Gande, Jigzhi, Madoi, and Maqin counties; Haibei Tibetan Autonomous Prefecture: Menyuan Hui Autonomous County; Hainan Tibetan Autonomous Prefecture: Xinghai County; Haixi Mongol and Tibetan Autonomous Prefecture: Tianjun County, Delingha, and Golmud county-level cities; Huangnan Tibetan Autonomous Prefecture: Jainca, Tongren, and Zeku counties; and Yushu Tibetan Autonomous Prefecture: Chindu, Nangchen, Qumalai, Zadoi, and Zhiduo counties, and also Yushu county-level city), northwestern Sichuan Province (Shiqu County), Tibet Autonomous Region (Chamdo prefecture-level city: Riwoche County; Lhasa prefecture-level city: Damxung County; Nagqu prefecture-level city: Amdo, Baingoin, and Shuanghu counties; and Shigatse prefecture-level city: Tingri County), and Xinjiang Uygur Autonomous Region (Ruoqiang County).

To the best of my knowledge, it is the *Iris* species found at the highest elevation. *Iris thoroldii* is found at elevations ranging from 3200 to 5400 m, or even up to 5800 m [41] (p. 537), above sea level and is adapted to a variety of environmental stresses such as extreme temperatures, drought, high winds, high UV radiation, and paucity of soil nutrients. There is every reason to consider it one of the extremely cold-hardy species in *I*. subgen. *Iris* (i.e., bearded irises) and, along with another such species *I. setosa* Pall. ex Link [72], in the genus.

To survive harsh high-elevation environments, *I. thoroldii* has evolved a number of morphological adaptations to achieve cold hardiness such as the dwarf habit, the vertical rhizome, the contractile adventitious roots, the narrow rosette leaves covered with a waxy coating that is aggregated into a dense tuft and protected from the outside by a few papery leaves, the significantly shortened flowering stem, the pedicel that does not emerge above ground, and the elongated perianth tube supported by surrounding, tightly arranged, and elongated bracts. The fruits are located close to the ground and, therefore, seeds are within the plant, which means that the main mode of seed dispersal is gravity (barochory; see https://ppbc.iplant.cn/tu/28427, accessed on 23 October 2023). Also, the aril attracts ants that are widespread on the Tibetan Plateau [73] and can be involved in the seed dispersal (myrmecochory). Furthermore, *I. thoroldii* shows remarkable flower color diversity, which contributes to its attractiveness for pollinators. In addition, it is likely that *I. thoroldii* has adapted to high-elevation habitats through establishing stable symbiosis with arbuscular mycorrhizal fungi [44].

The species is found in sandy, stony, gravelly, pebbly habitats, or on soft black soil in the Tibetan alpine meadows, alpine steppes, and desert steppes, in shady hillsides, on turfed or open slopes and hilltops, among rocks, on wind-eroded ridges, and also on sunny flood plains, gently sloping terraces and plateaus, in ravines, overgrazed areas, and sometimes in spruce forests and scrubs. The flowering time is May to July; the fruiting time is July to August.

## 5. Conclusions

The great number of species, abundance of synonyms, and taxonomic complexities have posed some difficulties in understanding the genus *Iris*, and, therefore, critical studies are needed to address them. The present taxonomic investigation will contribute to our knowledge of the Chinese species, in particular the identity of *I. thoroldii*, an endemic species to China distributed on the Tibetan Plateau and in adjacent areas, including central Gansu, Qinghai, and northwestern Sichuan provinces, and also Tibet and Xinjiang Uygur autonomous regions. *Iris thoroldii* has long been recognized as a taxonomic synonym of *I. potaninii*, with the latter, however, occurring in Russia (southern Siberia), Mongolia, and China (northeastern and western Inner Mongolia, northwestern Heilongjiang Province, and the northern Ningxia Autonomous Region). There is, accordingly, variation in the flower color of *I. thoroldii*, which has caused much confusion as to the taxonomic circumscription. Thus, depending on the flower color, the Tibetan plants are referred to as two species that are found growing together: those with yellow flowers are erroneously considered *I. potaninii*, and blue-flowered plants are considered *I. zhaoana*, a nomenclatural synonym of *I. potaninii* var. *ionantha.*

However, a detailed analysis of the protologue information, the existing literature on *Iris*, herbarium specimens (including original material), and images of living plants clearly indicate that the Tibetan plants are significantly different from *I. potaninii* in macromorphological characteristics and geographical distribution, and that *I. thoroldii* should be considered a distinct species. *Iris thoroldii* can be distinguished from *I. potaninii* on the basis of the shape of the rhizome, rosette and stem leaves, falls, standards, and aril, and also the length of the flowering stem, bracts, pedicel, and perianth tube. Furthermore, dry plants of *I. thoroldii* are always easily distinguishable from *I. potaninii* by the curled remnants of leaves.

The present study is a complete contribution to the taxonomy of *I. thoroldii*. To avoid further confusion, two forms of *I. thoroldii* are accepted here: the autonymic yellow-flowered form and the form with blue or purple color, *I. thoroldii* f. *ionantha*. I hope that the results obtained will not only be useful for understanding the nomenclature and taxonomy of *I. thoroldii*, but that they will also stimulate Chinese botanists to undertake more field efforts and study the *Iris* species in China. In particular, additional information on the chromosome number of *I. thoroldii* is needed.

It should also be emphasized that, inhabiting elevations up to 5800 m [41], *I. thoroldii* is the highest-elevation species in the genus *Iris* and possibly in Iridaceae. In this regard, it may be of interest as a potential source of physiologically active substances. As is known, many active derivatives from high-elevation plants show therapeutic potential [74]. Furthermore, *I. thoroldii* has evolved multiple ecological and reproductive strategies for adapting to the harsh environments of the Tibetan Plateau. For these reasons, it may be considered the perfect material for investigating the physiological capacity of plants at the upper limit of their distribution and the evolution of floral colors.

## Figures and Tables

**Table 1:** Comparative morphology of the *Iris* species studied.

No.	Characters	*I. thoroldii*	*I. potaninii*
1	Rhizome shape	Shortened, vertical	Shortened, horizontal, or vertical
2	Adventitious root shape	Contractile	Contractile
3	Rosette leaf shape	Narrow-linear	Narrow-linear
4	Rosette leaf apex shape	Obtuse or acute	Narrowly acute
5	Rosette leaf texture	Soft	Tough
6	Rosette leaf length	11.5 (4.5–28)	13 (5–29)
7	Rosette leaf width	0.3 (0.15–0.5)	0.3 (0.1–0.5)
8	Leaf remnants’ shape	Straight in wild, curled in dry	Straight
9	Leaf remnants’ height	4.8 (1.5–12)	2.8 (1–5)
10	Stem height	3.6 (1.5–6)	1.5 (0.5–2.5)
11	Number of stem leaves	2	2–3
12	Stem leaf length	3.6 (2–5.6)	4.5 (2.5–6)
13	Stem leaf shape	Lanceolate, apex acute	Lanceolate, apex acuminate
14	Number of bracts	2	2
15	Bract shape	Lanceolate, apex acute	Lanceolate, apex acute
16	Bract texture	Membranous	Membranous
17	Bract length	5 (2.2–8.5)	4.2 (2.5–6)
18	Pedicel length	0.7	0.1 (0–0.2)
19	Perianth tube length	5.3 (2.5–9.2)	3.3 (1.5–6)
20	Number of flowers	1	1
21	Flower color	Yellow, nearly white, blue, purple, or blue-purple	Yellow, pale yellow
22	Flower diameter	3.3 (2.2–5.5)	3.6 (2–5.5)
23	Blade of fall shape	Obovate	Lingulate
24	Fall length	3 (2.5–4.5)	3.5 (2–4.5)
25	Fall width	1.1 (0.7–1.7)	1.2 (0.6–1.6)
26	Standard shape	Oblanceolate	Obovate
27	Standard length	2.6 (2–3.2)	3 (2.4–3.5)
28	Standard width	0.8 (0.5–1.2)	1 (0.7–1.2)
29	Fruit shape	Elliptical or globose	Elliptical
30	Fruit length	2.1 (1.5–3)	2.8 (2–4)
31	Fruit width	1.5 (1–2)	1.5 (0.8–2)
32	Seed shape	Pyriform, with large white aril	Elliptical, with small beige aril
33	Seed color	Dark brown	Brown or dark brown
34	Seed length	0.4 (0.4–0.5)	0.4 (0.3–0.5)
35	Seed width	0.2 (0.2–0.3)	0.3 (0.2–0.3)

All measurements are in centimeters. Data are presented as mean (minimum–maximum). See Supplementary raw data in Table S1 for more details; for illustrations, see Figure 2 and Figure 4.

## Data Availability

All data supporting the reported results are presented as Supplementary Materials.

## Supplementary Materials

The following supporting information can be downloaded at: https://www.mdpi.com/article/10.3390/plants12223879/s1, Annex 1: Herbarium specimens of *Iris thoroldii* examined; Table S1: Raw data of the morphological analysis of the *Iris* species studied (the numbers of the morphological characters correspond to those in Table 1; all measurements are in centimeters).
