# Supplementary material for: Taxonomic Reinstatement of the Endemic Chinese Species Iris thoroldii (Iridaceae) from I. potaninii and Reassessment of I. zhaoana
Source: Plants (Basel). 2023 Nov 16;12(22):3879. doi: 10.3390/plants12223879 (PMC10674449; doi:10.3390/plants12223879)
Supplement: Supplementary file 1 [file plants-12-03879-s001.zip › Annex 1.pdf]

Annex 1. Herbarium specimens of *Iris thoroldii* examined.

(i) **Specimens of *I. thoroldii* deposited at LE** (https://herbariumle.ru/?t=occ&s=Iris%20thoroldii&f=%5Ball%5D, accessed on 23 October 2023).

The collections of N.M. Przhevalsky from his third and fourth journeys to Central Asia: China occidentalis, regio Tangut (prov. Kansu), [fl. yellow], 1880, *N.M. Przhevalsky s.n.* (LE01071969!; sub "*Iris potaninii* Maxim."); China occidentalis, regio Tangut (prov. Kansu), fauce fl. Baga-gorgi, 9500 p.s.m., ad …, [fl. yellow], 21 April/3 May 1880, *N.M. Przhevalsky 21* (LE01072829!); China occidentalis, regio Tangut (prov. Kansu), ad affl. fl. Baga-gorgi, in fauce inter …, [fl.], 27 April/9 May 1880, *N.M. Przhevalsky 46* (LE01072828!; sub "*Iris potaninii* Maxim."); Tibet borealis, [fl. blue], 1884, *N.M. Przhevalsky s.n.* (LE01071976!; sub "*Iris tigridia* Bge."); Fauce fl. Nomohungol ad rivulus inter lapidis parce, fl. pallide flavi, in *I.* spec. …, [fl.], 11/23 May 1884, *N.M. Przhevalsky s.n.* (LE01072827!); Fauce fl. Nomohungol, lecta ex terra selata nost frigis – 23°, fl. flavescentis, [fl.], 23 May/4 June 1884, *N.M. Przhevalsky s.n.* (LE01072826!); Valle fl. Nomohungol, fl. flavescentis, [fl.], 24 May/5 June 1884, *N.M. Przhevalsky s.n.* (LE01071977!); Tibet borealis, in arenosis humidis, 14000 ft., [fl. blue], 24 May/5 June 1884, *N.M. Przhevalsky s.n.* (LE00050571!); Tibet borealis, latis *I. divortii* fl. … et Yangtze, fl. flava, [fl. yellow and blue], 31 May/11 June 1884, *N.M. Przhevalsky s.n.* (LE01071970!); In montosis … fl. Dytschii, [fl. yellow and blue], 8/20 June 1884, *N.M. Przhevalsky s.n.* (LE01072825!); Ripa sinistra p. Yangtze (Dytschi) 1300 p.s.m., fl. pallide coerulei et flavescents, [fl.], 17/29 June 1884, *N.M. Przhevalsky s.n.* (LE00050572!).

The collection of V.I. Roborovsky from the Przhevalsky's fourth journey to Central Asia (viz. Qinghai Province): Kuen-Lun, Yuzhno Kukunor mountain range, southern slope, 11000 ft., on loess, flowers pale yellow, 6 May 1895, *V.I. Roborovsky 37* [originally in Russian] (LE01071972!); Kuen-Lun, upper Qaidam, Sarlyk-ula ridge, 11–12000 ft., on clay soil, flowers blue, 11 May 1895, *V.I. Roborovsky 45* [originally in Russian] (LE01071975!); Kuen-Lun, upper Qaidam, Sarlyk-ula ridge, northern slope, 11000 ft., flowers pale yellow, 20 May 1895, *V.I. Roborovsky 35* [originally in Russian] (LE01071973!, LE01071974!).

The collection of G.N. Potanin from his journey to Gansu Province, China: China borealis, prov. Kansu occidentali, ad rivulum Sonpa-Zonwa, infra Zabrun, [fl.], 20 May 1885, *G.N. Potanin s.n.* (LE01071992!; sub "*Iris tigridia* Bge."); China borealis, prov. Kansu occidentali, fl. flavi et coerulei, 28 May 1886, *G.N. Potanin s.n.* (LE01071968!; sub "*Iris potaninii* Maxim."); China borealis, prov. Kansu occidentali, declivitas N. trajectus Zagen-Daban, [fl. yellow and blue], 30 May 1886, *G.N. Potanin s.n.* (LE00050573!, LE01070703!; sub "*Iris potaninii* Maxim.").

The collection of V.F. Ladygin from the P.K. Kozlov journey to Tibet (with original labels in Russian): [Qinghai Province] Burhan Buda mountain range, northern slope, Nomokhun-gol gorge, alpine meadows, humus and clay-rocky soil, flowers yellow, 12000 ft., 18 May 1900, *V.F. Ladygin 20* (LE01071984!, LE01071988!, LE01071991!); Burhan Buda mountain range, northern slope, Nomokhun-gol gorge, 14000 ft., flowers dark purple, in alpine mountain zones (above the yellow iris under No. *20*), on humus, 18 May 1900, *V.F. Ladygin 27* (LE01071980!, LE01071987!); Tibet, Lake Alyk-Nor, *Iris* on open loose clay areas, 12–13000 ft., 30 May 1900, *V.F. Ladygin s.n.* (LE01072830!); Burhan Buda mountain range, southern slope, riverbeds and mountainsides, on clay and clayey-stony loose soil, 13500–14000 ft., [fl. blue], 30 May 1900, *V.F. Ladygin s.n.* (LE01071985!); Tibet, Russkoye Lake, Dokagyn-gol, 13–14000 ft., flowers lilac-purple lilac-purple or more often dirty gray, 15 June 1900, *V.F. Ladygin s.n.* (LE01071990!); Gansu, [fl. yellow and blue], 1901, *V.F. Ladygin s.n.* (LE01071971!, LE01071979!); [Xizang] Kham (Tibet), Yang-tze-kiang basin, Om-bun-da tract, on the Yalong Jiang river, on clayey open areas along the valley bottom, flowers yellow, fragrant, 12300 ft., 6 May 1901, *V.F. Ladygin 34* (LE01071978!, LE01071986!); Kham (Tibet), Yang-tze-kiang basin, Tzergen Tzergen

tract, on the sunny side of the mountain and along the bottom of the gorge, clayey-stony soil and humus, dark purple flowers, fragrant, 13500 ft., 14 May 1901, *V.F. Ladygin 42* (LE01071982!, LE01071983!); Kham (Tibet), Yang-tze-kiang basin, Gorin-Chyu River valley, on clay and humus, flowers purple and light purple, 14000 ft., 17 May 1901, *V.F. Ladygin s.n.* (LE01071981!); Tibet, the Yellow River Basin, the upper Serg-Chyu river, spreads into a circle or ring on clay soil, flowers nearly white, 14000 ft., 23 May 1901, *V.F. Ladygin s.n.* (LE01071989!).

(ii) **Digital specimens available in the Chinese Virtual Herbarium database are labelled *I. thoroldii*, *I. potaninii*, and *I. potaninii* var. *ionantha*** (https://www.cvh.ac.cn/index.php, accessed on 23 October 2023) **and those available in the National Specimen Information Infrastructure database are labelled *I. potaninii*** (indicated by asterisk; see http://www.nsii.org.cn/2017/home.php, accessed on 23 October 2023). The list includes information on elevation, collection data, collection number, and herbarium codes (for the specimens deposited at HNWP, except for one specimen, inventory numbers are indicated).

Qinghai Province: 06.1962, *41-01* (PE01013067); 4240 m, 15.06.1959, *453* (HNWP No. 002938, PE01013110); 4240 m, 15.06.1959, *454* (HNWP No. 002939, PE01013064); 3700 m, 26.07.1959, *643* (PE01013066); 08.06.1960, *021* (LZD0003150); 3800 m, 30.06.1960, *2825* (PE01013065); 4700 m, 14.06.1963, *10007* (HNWP No. 002930, HNWP No. 27724); 4700 m, 14.06.1963, *10008* (HNWP No. 002926, HNWP No. 27725); 3800 m, 02.06.1964, 00006 (HNWP No. 002924, PE01803390); 3800 m, 26.05.1965, *00836* (HNWP No.0218509, PE01013109, PE01803388); 5000 m, 29.05.1965, *00131* (HNWP No. 13437, PE01803387); 4300 m, 05.06.1965, *002* (HNWP No. 0218511, PE01803391); 4600 m, 14.06.1965, *00930* (HNWP No. 12537); 3950 m, 19.06.1965, *0037* (HNWP No. 11969, HNWP No. 0236923); 3950 m, 19.06.1965, *17* (NAS00555428); 3950 m, 19.06.1965, *0017* (HNWP No. 11949); 4500 m, 24.05.1966, *5* (HNWP No. 17992); 26.05.1966, *27* (HNWP No. 17804); 4100 m, 01.06.1966, *00580* (HNWP No. 17235); 4600 m, 04.06.1966, *00589* (HNWP No. 17244); 3420 m, 08.06.1966, *14* (HNWP No. 0218532); 3420 m, 08.06.1966, *16* (HNWP No. 21081); 4190 m, 09.06.1966, *48* (HNWP No. 18033); 4600 m, 23.06.1966, *0026* (HNWP No. 17635); 3450 m, 13.06.1970, *684* (HNWP No. 0218512, HNWP No. 131595, HNWP No. 21942); 3950 m, 06.06.1974, *0020* (HNWP No. 0218520); 3750 m, 06.06.1974, *20* (HNWP No. 179887); 4680 m, 13.06.1975, *75-0255* (PE01013126, PE01013127); 4680 m, 13.06.1975, *0256* (HNWP No. 53427, PE01013081, PE01013082); 3250 m, 11.07.1975, *11265* (HNWP No. 46829); 3600 m, 10.08.1975, *12061* (HNWP No. 0218513); 3200 m, 30.05.1976, *008* (HNWP No. 57610); 3200 m, 30.05.1976, *006* (HNWP No. 57608); 4300 m, 11.06.1977, *028* (HNWP No. 120386); 4300 m, 23.06.1977, *64* (HNWP No. 120385); 4200 m, 23.06.1977, 028 (HNWP No. 110668); 3610 m, 15.07.1977, *2345* (HNWP No. 63459); 4700 m, 07.1978, *038* (HNWP No. 77598); 4000 m, 06.06.1981 (HNWP No. 99198); 4300 m, 04.07.1981, *094* (HNWP No. 101288); 3820 m, 05.07.1981, *164* (HNWP No. 101671); 4400 m, 08.06.1985, *0063* (HNWP No. 109477); 4400 m, 08.06.1985,*0064* (HNWP No. 109478); 21.06.1988, *25* (HNWP No. 0231278); 3500 m, 12.08.1988, *1081* (HNWP No. 151628); 5100 m, 30.08.1989, *CG89-310* (HNWP No. 155251, HNWP No. 155252); 4300 m, 28.05.1990, *k-006* (PE02045215); 3800 m, 30.05.1990, *k-608* (HNWP No. 164845); 4950 m, 07.06.1990, *R-015* (HNWP No. 0218510); 4900 m, 08.06.1990, *K-616* (HNWP No. 164841, KUN1223396); 4900 m, 08.06.1990, *K-617* (KUN1223402); 5100 m, 10.06.1990, *K-022* (KUN1223400); 4900–5100 m, 10.06.1990, *K-623* (KUN1223397); 4750 m, 15.06.1990, *K-034* (KUN1223401, HNWP No. 164852); 4750 m, 15.06.1990, *K-035* (HNWP No. 164843, KUN1223395); 4900 m, 17.06.1990, *K-650* (HNWP No. 164844, KUN1223399); 4730 m, 18.06.1990, *K-655* (KUN1223404); 4580 m, 18.06.1990, *k-32* (PE02048961); 4820 m, 22.06.1990, *K-072* (HNWP No. 164842, KUN1223398); 4820 m, 22.06.1990, *K-073* (KUN1223403, HNWP No. 164847); 4700 m, 15.06.1993, *17138* (HNWP No. 184094); 4050–4300 m, 10.08.1993, *1072* (HNWP No. 0226608, HNWP No. 0226609, HNWP No. 172346, PE01013068, PE01013069); 4400–4700 m, 15.08.1993, *1263* (HNWP No. 0221344,

HNWP No. 0232237, PE01013070); 4400 m, 27.05.1999, *k-607* (HNWP No. 164846); 4800 m, 06.06.1999, *16979* (HNWP No. 184393); 5200 m, 07.06.1999, *16991* (HNWP No. 184381); 4700 m, 09.06.1999, *17042* (HNWP No. 184276); 4900 m, 10.06.1999, *17049* (HNWP No. 184229); 4700 m, 11.06.1999, *17057* (HNWP No. 184217, HNWP No. 184218); 5000 m, 14.06.1999, *17128* (HNWP No. 184107, HNWP No. 184108); 5000 m, 15.06.1999, *17134* (HNWP No. 184099, HNWP No. 184100); 4700 m, 15.06.1999, *17137* (HNWP No. 184095, HNWP No. 184096); 4600 m, 16.06.1999, *17140* (HNWP No. 184090, HNWP No. 184091); 4600 m, 16.06.1999, *17141* (HNWP No. 184089); 4700 m, 18.06.1999, *17142* (HNWP No. 184087); 4700 m, 18.06.1999, *17143* (HNWP No. 184085, HNWP No. 184086); 4680 m, 18.06.1999, *17144* (HNWP No. 184083, HNWP No. 184084); 4600 m, 18.06.1999, *17145* (HNWP No. 184082); 20.07.2000, *907* (HNWP No. 190307); 20.07.2000, *909* (HNWP No. 190304, HNWP No. 190305); 4170 m, 20.07.2004, *28931* (HNWP No. 0218514, HNWP No. 199059); 4600 m, 29.07.2004, *30386* (HNWP No. 198456); 4300 m, 04.07.2005, *32527* (HNWP No. 0210576, HNWP No. 0210577); 4240 m, 12.07.2014, *QH2014692* (BNU0032091); 4700 m, 06.08.2014, *2068* (BJFC00080855); 4320 m, 19.07.2016, *7124* (BNU0030727); 3171 m, 16.06.2018, *ZXX18114* (KUN1438281).

Tibet Autonomous Region: 3500 m, 25.05.1926, *13964* (IBSC0628886, PE00034032); 4700 m, 03.06.1960, *168* (PE01013124); 4600 m, 06.06.1960, *188* (PE01013085); 4700 m, 17.06.1961, *3417* (PE01013125, PE01803381, PE01803382); 4700 m, 17.06.1961, *3425* (PE01013083, PE01803398, PE01803399); 4700 m, 04.08.1961, *3703* (PE01803393, PE01013084); 4700 m, 14.06.1963, *1873* (HNWP No. 57132, KUN0360486, LZD0003148; PE02238449, sub *I. loczyi* Kanitz); 4700 m, 14.06.1963, *1874* (LZD0003149, HNWP No. 57133, KUN0360485, KUN0360485); 4700 m, 18.06.1966, *4130* (PE01013116, PE01013117); 4950 m, 20.06.1966, *4326* (PE01013114, PE01013115); 4400 m, 28.05.1975, *7401* (PE01803386); 4400 m, 05.06.1975, *014* (PE01013112, PE01013113); 4300 m, 06.06.1975, *6433* (PE01485384, PE01803384, PE01803385); 5050 m, 04.07.1975, *4523* (PE01803394); 3800 m, 15.05.1976, *8024* (PE01803383); 4800–5000 m, 05.06.1976, *11698* (PE01803380, PE02083282, PE02083283); 4800–5000 m, 05.06.1976, *11699* (PE01803395, PE02083284, PE02083285); 4700 m, 08.06.1976, *10475* (HNWP No. 59725, PE01013119); 4500 m, 10.06.1976, *10498* (HNWP No. 59051, PE01013073, PE01803396); 5300 m, 10.06.1976, *10506* (PE01013118); 4900–5000 m, 10.06.1976, *9422* (HNWP No. 60661, PE01013075, PE01013076); 4900–5000 m, 10.06.1976, *9424* (HNWP No. 60663, PE01013122, PE01013123); 4700 m, 20.06.1976, *10517* (HNWP No. 59055, PE01013074, PE01803397); 5100–5300 m, 23.06.1976, *9492* (HNWP No. 59417, PE01013120, PE01013121); 5100–5200 m, 23.06.1976, *9493* (HNWP No. 59416, PE01013077, PE01013078); 4900 m, 31.07.1976, *10702* (PE01013072); 4700–5000 m, 05.08.1976, *10824* (HNWP No. 58062, PE01803392, PE01013071); 5000 m, 17.08.1976, *10016* (HNWP No. 61084, PE01013079, PE01013080); 3700 m, 07.05.1986, *709* (PE01013130); 4700–4900 m, 24.05.1986, *1356* (PE01013128, PE01013129); 4600 m, 14.08.1988, *2705* (HNWP No. 149641, HNWP No. 150663); 3500 m, 02.06.1990, *T218* (PE01013111); 4800 m, 26.08.1990, *90-411* (IBSC0628887); 28.07.2009, *0490* (HNWP No. 268597*); 4707 m, 21.08.2009, *YangYP-Q-2145* (KUN1393266); 4740 m, 24.06.2018, *1-492* (HNWP00023928).

Xinjiang Uygur Autonomous Region: Ruoqiang County, s.d., s.n. (XJA00066082*, XJA00066083*, XJA00066087*); 3900–4100 m, 30.06.1982, *4* (XJA00066081*); 4150 m, 21.06.1988, *88013* (XJA00066079*); 4150 m, 21.06.1988, *88014* (XJA00066080*, XJA00066088*); 4300 m, 21.08.1988, *1641* (HNWP No. 150744); 4000 m, 04.06.2012, *XJ048* (BNU0012973); 29.06.2013, *XJ0101* (BNU0022618*); 3980 m, 24.07.2014, *ALT.027* (BNU0027560); 4126 m, 28.07.2014, *ALT.167* (BNU0027559).
